# A Gene Selection Method for Cancer Classification

**DOI:** 10.1155/2012/586246

**Published:** 2012-11-20

**Authors:** Xiaodong Wang, Jun Tian

**Affiliations:** ^1^Faculty of Mathematics and Computer Science, Fuzhou University, Fuzhou 350002, China; ^2^School of Public Health, Fujian Medical University, Fuzhou 350004, China

## Abstract

This paper proposes a method to select a set of genes from a large number of genes with the ability of classifying types of diseases. The proposed gene selection method is designed according to correlation analysis and the concept of 95% reference range. The method is very simple and uses the information of all genes. We have used the method in leukemia patients and achieved good classification results.

## 1. Introduction

In the clinical treatment of cancer, the corresponding treatment methods and measures are based on the correct classification of tumors. The traditional classification methods are based on tumor cell morphology, but sometimes tumors with the same histopathological type have different responses to treatment. So it becomes the current hot research topic to classify the tumor type using genomics [1, 2].

The gene chip experimental technology has provided a strong technology platform for tumor classification in genomics. By the discrimination analysis of gene expression levels in patients with different types of disease, the discrimination function established can be used to assist classification of clinical cases [3, 4].

As the gene chips have a very large number of genes, not all of these genes will provide information on the classification of type. When the expression levels of genes in different types of tissue samples do not change much, these genes have statistically no or only small discrimination capability. These genes are redundant.

Excluding these genes without classification capabilities will help to optimize the gene discrimination function which will be convenient for practical use. Therefore, it is necessary to select the genes with classification capabilities from a large number of genes [5].

Stepwise discrimination analysis is a commonly used statistical method for variable selection. However, for tissue samples containing thousands of genes, the stepwise discrimination analysis module in commonly used statistical software packages such as SAS and SPSS cannot function properly.

This paper presents a gene screening method that can analyze the classification capabilities of genes from thousands of genes and select the genes helpful to gene classification. The present new method has achieved good results in practice.

In the following sections we describe our studies on the statistical method for gene screening with discriminating ability in tumor classification.

In Methods we describe our new method for gene screening.

In Results we provide an application of our method in the study of the classification of leukemia patients.

Some concluding remarks are presented in Discussion.

## 2. Methods

Let a kind of disease have two subtypes *A* and *B*. There are in total *m* cases of the disease. Of these, *n*_1_ cases are type *A* and *n*_2_ cases are type *B*. One tumor tissue sample was obtained from each of the patients. The expression levels of the *n* genes *g*_1_, *g*,…, *g*_*n*_ of each tissue sample were detected by gene chip.

### 2.1. The Correlation Coefficients Computation for Each Gene and the Classification Vector

If we list the *n*_1_ cases of type *A* first and the *n*_2_ cases of type *B* come in the tail, then the corresponding classification vector will be *c* = (*c*_1_, *c*_2_,…, *c*_*m*_), where *c*_*j*_ corresponds to the case *j*. If the case *j* is of type *A*, then *c*_*j*_ = 1, otherwise *c*_*j*_ = 0, *j* = 1,2,…*m*. Therefore, the classification vector has the form $c=(\overset{n_{1}}{\overset{︷}{1,1,\ldots 1}},\overset{n_{2}}{\overset{︷}{0,0,\ldots ,0)}}$.

Let the mean and standard deviation of the score for gene *g*_*i*_ in the *n*_1_ tissue samples of type *A* be *μ*_*A*_(*g*_*i*_) and *σ*_*A*_(*g*_*i*_), respectively. Similarly, the mean and standard deviation of the score for gene *g*_*i*_ in the *n*_2_ tissue samples of type *B* will be *μ*_*B*_(*g*_*i*_) and *σ*_*B*_(*g*_*i*_).

The correlation coefficient for gene *g*_*i*_ and the classification vector *c* is defined as

(1) $$\begin{array}{c} P\left(g_{i},c\right)=\frac{\mu_{A}\left(g_{i}\right)-\mu_{B}\left(g_{i}\right)}{\sigma_{A}\left(g_{i}\right)+\sigma_{B}\left(g_{i}\right)}. \end{array}$$

The greater the absolute value of *P*(*g*_*i*_, *c*), the stronger the correlation of gene *g*_*i*_ and the classification vector *c*. In other words, the gene *g*_*i*_ has the ability to distinguish between type *A* and type *B*.

From formula (1) we can compute *P*(*g*_1_, *c*), *P*(*g*_2_, *c*),…, *P*(*g*_*n*_, *c*) for genes *g*_1_, *g*,…, *g*_*n*_.

### 2.2. Determine the Critical Value of Gene Screening

Let *c*_1_*, *c*_2_*,…, *c*_n_* be *n* random permutation vectors of the classification vector *c* = (1,…, 1,0,…, 0).

We now perform the following three steps of computation for each random permutation vector *c*_j_*, *j* = 1,2,…, *n*.

(1) Collect the first *n*_1_ cases in *c*_j_* to a set denoted as class_1_ and the remaining cases in *c*_j_* to a set denoted as class_2_.
(2) For *i* = 1,2,…, *n*, compute *μ*_class_1__(*g*_*i*_, *c*_j_*) and *σ*_class_1__  (*g*_*i*_, *c*_j_*) corresponding to *g*_*i*_ in class_1_ and *μ*_class_2__(*g*_*i*_, *c*_j_*) and *σ*_class_2__(*g*_*i*_, *c*_j_*) corresponding to *g*_*i*_ in class_2_, respectively.
(3) For *i* = 1,2,…, *n*, compute
  (2) $$\begin{array}{c} P\left(g_{i},c_{j}^{*}\right)=\frac{\mu_{{\text{class}}_{1}}\left(g_{i},c_{j}^{*}\right)-\mu_{{\text{class}}_{2}}\left(g_{i},c_{j}^{*}\right)}{\sigma_{{\text{class}}_{1}}\left(g_{i},c_{j}^{*}\right)+\sigma_{{\text{class}}_{2}}\left(g_{i},c_{j}^{*}\right)}. \end{array}$$

From the computation above, we obtain the correlation coefficients for the *n* gene expression levels and the *n* random permutation vector *c*_j_*, *j* = 1,2,…, *n* as follows:

(3) $$\begin{array}{c} \left(\begin{array}{cccc} P\left(g_{1},c_{1}^{*}\right) & P\left(g_{1},c_{2}^{*}\right) & \cdots & P\left(g_{1},c_{n}^{*}\right)\\ P\left(g_{2},c_{1}^{*}\right) & P\left(g_{2},c_{2}^{*}\right) & \cdots & P\left(g_{2},c_{n}^{*}\right)\\ \vdots & \vdots & \vdots & \vdots \\ P\left(g_{n},c_{1}^{*}\right) & P\left(g_{n},c_{2}^{*}\right) & \cdots & P\left(g_{n},c_{n}^{*}\right) \end{array}\right). \end{array}$$

For a given value *r* and vector *c*, denote the number of genes having correlation coefficients not less than *r* as *N*_1_(*c*, *r*) and the number of genes having correlation coefficients not greater than −*r* as *N*_2_(*c*, *r*).

For all 1 ≤ *i*, *j* ≤ *n*, we can define the following formulas:

(4) $$\begin{array}{c} S_{1}\left(c_{j}^{*},g_{i},r\right)=\{\begin{array}{c} \begin{array}{cc} 1 & P\left(g_{i},c_{j}^{*}\right)\geq r \end{array}\\ \begin{array}{cc} 0 & P\left(g_{i},c_{j}^{*}\right)<r \end{array}, \end{array} \end{array}$$

(5) $$\begin{array}{c} N_{1}\left(c_{j}^{*},r\right)=\sum_{i=1}^{n}S_{1}\left(c_{j}^{*},g_{i},r\right), \end{array}$$

where *N*_1_(*c*_j_*, *r*) is the number of genes having correlation coefficients with vector *c*_j_* not less than *r* and *N*_2_(*c*_j_*, *r*) is the number of genes having correlation coefficients with vector *c*_j_* not greater than −*r*, *j* = 1,2,…, *n*.

The right 5% quantile of the *n* items *N*_1_(*c*_1_*, *r*), *N*_1_(*c*_2_*, *r*),…*N*_1_(*c*_n_*, *r*) is denoted as *L*_1_^*r*^ and the right 5% quantile of the *n* items *N*_2_(*c*_1_*, *r*), *N*_2_(*c*_2_*, *r*),…*N*_2_(*c*_n_*, *r*) is denoted as *L*_2_^*r*^.

By increasing the value of *r* gradually we can get

(6) $$\begin{array}{c} \left(\begin{array}{cccc} r_{1} & r_{2} & \cdots & r_{k}\\ N_{1}\left(c,r_{1}\right) & N_{1}\left(c,r_{2}\right) & \cdots & N_{1}\left(c,r_{k}\right)\\ L_{1}^{r_{1}} & L_{1}^{r_{2}} & \cdots & L_{1}^{r_{k}} \end{array}\right). \end{array}$$

**Figure 1:**
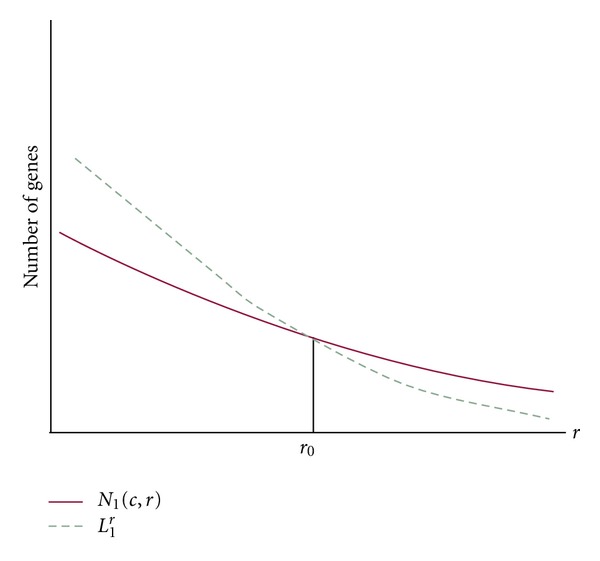
Two curves of (*r*_*i*_, *N*_1_(*c*, *r*_*i*_)) and (*r*_*i*_, *L*_1_^*r*_*i*_^).

If we draw two curves of (*r*_*i*_, *N*_1_(*c*, *r*_*i*_)) and (*r*_*i*_, *L*_1_^*r*_*i*_^) on the plane, we can see they have one intersection. The abscissa of the intersection is denoted as *r*_0_ (see Figure 1).

Similarly, for each *r*_*i*_ we also have

(7) $$\begin{array}{c} \left(\begin{array}{cccc} r_{1} & r_{2} & \cdots & r_{k}\\ N_{2}\left(c,r_{1}\right) & N_{2}\left(c,r_{2}\right) & \cdots & N_{2}\left(c,r_{k}\right)\\ L_{2}^{r_{1}} & L_{2}^{r_{2}} & \cdots & L_{2}^{r_{k}} \end{array}\right). \end{array}$$

**Figure 2:**
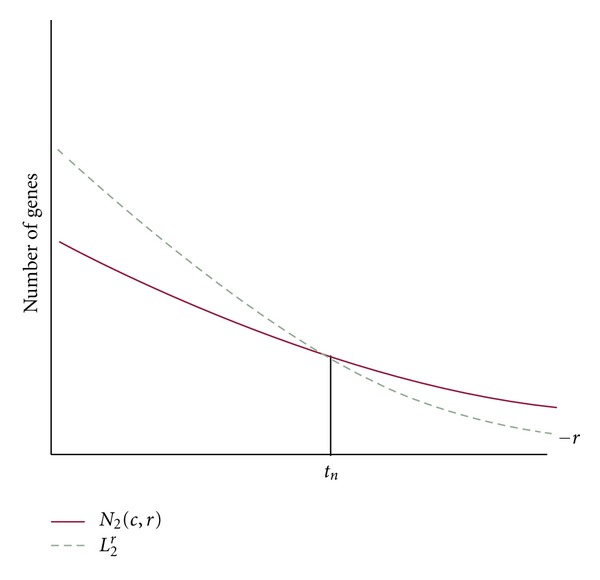
Two curves of (*r*_*i*_, *N*_2_(*c*, *r*_*i*_)) and (*r*_*i*_, *L*_2_^*r*_*i*_^).

If we draw two curves of (*r*_*i*_, *N*_2_(*c*, *r*_*i*_)) and (*r*_*i*_, *L*_2_^*r*_*i*_^) on the plane, we can see they have one intersection. The abscissa of the intersection is denoted as *t*_0_ (see Figure 2).

Let r* = max⁡{*r*_0_, |*t*_0_|}. If |*P*(*g*_*i*_, *c*)| ≥ r*, then gene *g*_*i*_ is considered to have the ability to distinguish between type *A* and type *B*. Therefore, it can be used as the index of the discrimination function for all 1 ≤ *i*, *j* ≤ *n*.

## 3. Results

We have applied our method in the study of the classification of leukemia patients. We obtained a 38 × 7129 data matrix by taking tissue samples from 38 cases of clinically diagnosed leukemia patients and 7129 gene expression levels being detected for tissue samples of each case. Of the 38 cases, 27 cases had been diagnosed as acute lymphoblastic leukemia (ALL) and 11 cases are acute myeloid leukemia (AML). Before the two-type discrimination analysis, the 7129 genes are screened first using the method presented in this paper.

There are 38 components in the classification vector *c* = (1,…, 1,0,…, 0), whichrepresentsthe original classificationvector. The first 27 components of *c* are 1 and the last 11 components of *c* are 0. The correlation coefficients of the expression level for each gene and the classification vector *c* are computed by formula (1).  Table 1 is the frequency distribution table for the 7129 absolute values of the correlation coefficients.

We generate 500 random permutation vectors *c*_j_* (*j* = 1,2,…, 500) by 500 times of random permutation of the vector *c* = (1,…, 1,0,…, 0). The correlation coefficients *P*(*g*_*i*_, *c*_j_*) of the expression level for each gene and the classification vector *c*_j_* are computed by formula (1) (1 ≤ *i* ≤ 7129, 1 ≤ *j* ≤ 500).

For the 6 values of *r* between 0.1 ~ 0.6 and all *j* (1 ≤ *j* ≤ 500), we compute *N*_1_(*c*_j_*, *r*) and *N*_2_(*c*_j_*, *r*) by formula (5) then their right 5% quantiles *L*_1_^*r*^ and *L*_2_^*r*^. The computation results are shown in Tables 2 and 3.

**Figure 3:**
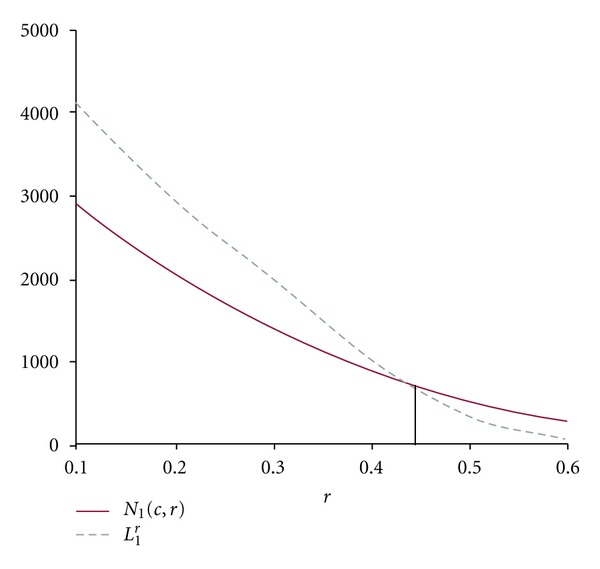
Curves of (*r*_*i*_, *N*_1_(*c*, *r*_*i*_)) and (*r*_*i*_, *L*_1_^*r*_*i*_^) for different value of *r*.

**Figure 4:**
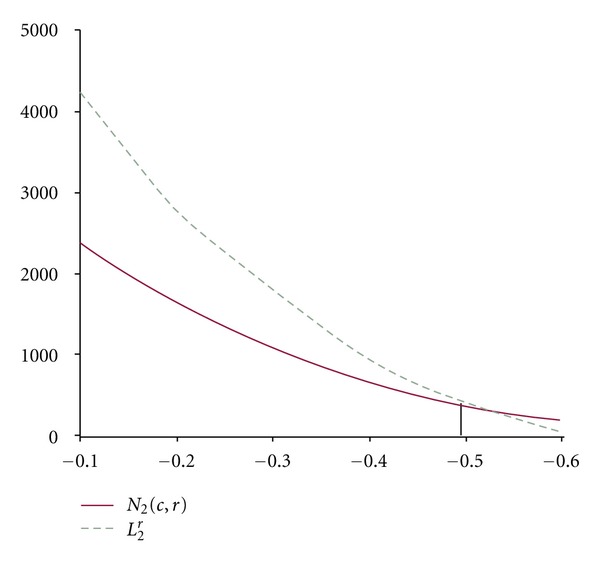
Curves of (*r*_*i*_, *N*_2_(*c*, *r*_*i*_)) and (*r*_*i*_, *L*_2_^*r*_*i*_^) for different value of *r*.

From the data of Tables 2 and 3, we can draw the corresponding curves as shown in Figures 3 and 4.

From Figures 3 and 4 we can read *r*_0_ = 0.44 and *t*_0_ = −0.49.

Therefore, r* = max⁡{0.44,0.49} = 0.49 ≈ 0.5.

There are in total 893 genes satisfying |*P*(*g*_*i*_, *c*)| ≥ 0.5.

By a two-type discrimination analysis for the tissue samples of the 38 leukemia patients using the 893 gene expression levels as variables, we can build a discrimination function. The 38 patients were identified and classified by using the discrimination function (discrimination function retrospective assessment). The miscarriage of justice was 0.

We have established a prospective evaluation of the discrimination function.

The data are taken from the website of the Broad Institute of MIT [6]. There are total 34 cases of leukemia patients (of which 20 cases of ALL and 14 cases of AML). The 893 gene data were substituted into the discrimination function and classified by type. The miscarriage of justice was 0.02.

Based on the above assessment, we believe the discrimination function established by selecting 893 genes with distinguishing capability from the 7129 genes using our method can be a good discrimination function for classifying leukemia patients and it will provide a good reference for the effective treatment.

## 4. Discussion

In the statistical methods of classification, the stepwise discrimination analysis is mainly used for variable selection. As the number of data in gene microarrays can be very large, the stepwise discrimination analysis module in commonly used statistical software packages such as SAS and SPSS would not function properly. We have tried to filter genes with classification ability for the whole sample of 7129 genes of the 27 cases of lymphoblastic leukemia and the 11 cases acute myeloid leukemia. The computer program crashed when a discriminant analysis or principal component analysis method was applied since the number of genes was too large. Therefore, we cannot perform discriminant analysis or principal component analysis for the data set on a personal computer.

Therefore, on such a large number of gene chip data for screening, using stepwise discrimination analysis to filter genes with classification ability in a personal computer is infeasible.

A common solution to this problem is to divide the large number of gene data into several groups of genes. The genes with classification ability in each group are selected by the stepwise discrimination analysis of the gene expression levels within each group. Finally, these discrimination functions of each group are combined to build a discrimination function for the whole of genes.

However, this method is also inadequate because the links between genes are separated artificially by gene group division. As tumors are diseases with multigene combined effects, separating the links between genes will reduce the classification ability of the final selected genes. It will in turn affect the subsequent analysis of the classification accuracy on new samples and the results are also not easy to explain [7].

In addition, how many groups of genes are to be divided into is also subjective and this will directly affect the final result for screening of the genes.

The principle idea behind our random permutation vectors method is very similar to a statistical approach, called Randomization Test [8], which is widely used in many applications. The application of the method implies that we have to enumerate all possible combinations of the elements in vector *c* and this is often a very difficult task. In the cases of this paper, there are total 38!/(27!×11!) = 1203322288 different combinations if we divide the 38 cases of leukemia patients into two groups of 27 and 11 cases, respectively. This huge number of combinations is really a restriction for us to apply the Randomization Test method to our cases. Therefore, we use the Monte Carlo sampling method further to the vector *c* to generate 500 random combinations. These 500 combinations are 500 random samples of all possible combinations of elements in vector *c*. Although the results of 500 samples do not produce an exact answer, it can be close to the exact answer [9]. In order to make the results closer to the exact answer, we may increase the number of random samples. For example, we may increase the number of random samples to 1000 or more in our cases.

Our presented method applies to selecting the genes with the ability of classifying types of diseases from a large number of genes. Compared to the stepwise discrimination analysis by groups, the new method has an obvious advantage of the full usage of information of all genes.

The new method has a low computational complexity and is very practical in practice. The main costs of computation are in the correlation coefficient computation when we need a random permutation vector from the vector *c* and this is not a difficult task for common personal computers.

In the real analysis circumstance, if there are too many genes with their absolute value greater than r*, then the value of r* can be adjusted to r* + *a*. The value of *a* can be adjusted according to the actual situation. The feasibility of the adjusted value of *a* can be checked by a retrospective assessment of the discrimination function established on the selected genes.

## Figures and Tables

**Table 1:** The frequency distribution table for the 7129 correlation coefficients.

*P*(*g*_*i*_, *c*)	Frequency	Percentage (%)	Cumulative percentage (%)
0.0~	1833	25.7	25.7
0.1~	1610	22.6	48.3
0.2~	1239	17.4	65.7
0.3~	941	13.2	78.9
0.4~	613	8.6	87.5
0.5~	411	5.8	93.2
0.6~	234	3.3	96.5
0.7~	131	1.8	98.4
0.8~	62	0.9	99.2
0.9~	55	0.8	100.0

Total	7129	100.0	—

**Table 2:** The right 5% quantiles of *N*_1_(*c*_j_*, *r*) (1 ≤ *j* ≤ 500).

*r*_*i*_	*N*_1_(*c*, *r*_*i*_)	*L*_1_^*r*_*i*_^
0.10	2907	4128
0.20	2058	2946
0.30	1385	1985
0.40	868	996
0.50	514	325
0.60	278	80

**Table 3:** The right 5% quantiles of *N*_2_(*c*_j_*, *r*) (1 ≤ *j* ≤ 500).

*r*_*i*_	*N*_2_(*c*, *r*_*i*_)	*L*_2_^*r*_*i*_^
−0.10	2389	4245
−0.20	1628	2725
−0.30	1062	1785
−0.40	638	902
−0.50	379	372
−0.60	204	53
